# Fundus-to-Antrum Ratio Measured with Fluoroscopy within One Week after Endoscopic Sleeve Gastroplasty Predicts Total Body Weight Loss over Time

**DOI:** 10.3390/jcm13133933

**Published:** 2024-07-04

**Authors:** Kaveh Hajifathalian, Kamal Amer, Dema Shamoon, Donevan Westerveld, Louis Aronne, Amit Mehta, Angela Wong, Grace Lo, Sarah Oh, Andrea Siobhan Kierans, Kamal M. Hassan, Ali Lahooti, Reem Z. Sharaiha

**Affiliations:** 1 Division of Gastroenterology and Hepatology, Rutgers New Jersey Medical School, University Hospital, Newark, NJ 07103, USA; 2 Division of Gastroenterology and Hepatology, St. Michael’s Medical Center, 111 Central Avenue, Newark, NJ 07102, USA; 3 Division of Gastroenterology and Hepatology, Weill Cornell Medicine, New York-Presbyterian Hospital, New York, NY 10065, USAkah4004@med.cornell.edu (K.M.H.);; 4 Division of Endocrinology Diabetes and Metabolism, Weill Cornell Medicine, New York-Presbyterian Hospital, New York, NY 10065, USA

**Keywords:** obesity, gastroplasty, bariatrics, endoscopic suturing, endoscopic sleeve gastroplasty

## Abstract

**Background and Aims**: Endoscopic sleeve gastroplasty (ESG) is a minimally invasive bariatric procedure to induce weight loss through restrictive physiology. This study was designed to evaluate the fluoroscopic measurement of gastric dimensions after ESG as a predictor of Total Body Weight Loss (TBWL) over time. **Methods**: Post-ESG patients were enrolled prospectively between August 2013 and August 2019. An upper gastrointestinal (GI) fluoroscopy was obtained within 7 days after the procedure. Two blinded, independent radiologists reviewed fluoroscopic images and measured the gastric lumen transverse diameter in three separate areas of the fundus, body, and antrum. The primary outcome was achieving a TBWL of ten percent or more after ESG. **Results**: In total, 162 patients were included in the analysis (65% female) and had a mean body mass index (BMI) of 39 ± 6 at baseline. Patients had a mean maximum TBWL of 16.5 ± 8.3%. Respectively, 92%, 75%, and 50% of patients achieved a TBWL of 5%, 10%, or 15% or more. The mean post-procedural UGI gastric fundus/antrum transverse measurement ratio was 1.2 ± 0.6. A higher fundus-to-antrum ratio was significantly associated with a TBWL of 10% or more during follow-up in the multivariable model (OR 2.49, 95% CI 1.31–4.71; *p*-value 0.005). The prediction score based on the fundus-to-antrum ratio hd an area under the ROC curve of 0.79 (95% CI 0.75–0.83) for predicting a TBWL of 10% or more during follow-up. **Conclusions**: Measuring gastric the fundus/antrum ratio within one week of endoscopic sleeve gastroplasty (ESG) is a consistent and independent predictive measure of sustained TBWL during long-term follow-up.

## 1. Introduction

Obesity is a global health concern associated with significant morbidity and mortality among men and women, all racial and ethnic groups, and all ages [1]. The management of obesity based on lifestyle modifications and pharmacologic approaches rarely leads to sustained weight loss [2]. Bariatric surgery is superior to lifestyle changes due to the reduction in weight and sustained weight loss, as well as the resolution of metabolic comorbidities in up to 80% of patients; however, costs, relatively increased risks, and limited access have decreased the eligibility of patients electing to pursue it [3].

Endoscopic sleeve gastroplasty (ESG) is a minimally invasive endoscopic approach to perform a gastric-restrictive bariatric procedure. In the literature, a mean total body weight loss of 15 to 20% after ESG has been reported [4]. Recent randomized clinical trials further substantiate these findings, demonstrating significant weight loss and improvements in metabolic comorbidities in individuals undergoing ESG with lifestyle modifications, compared to those adhering to lifestyle modifications alone [5]. Prior studies have also demonstrated the durability of these results with a mean total weight loss of 16% maintained at five years after the procedure [6]. Furthermore, previous studies have demonstrated a reduction in systolic blood pressure (SBP), hemoglobin A1c (HgA1c), serum triglycerides (STg), insulin resistance, and alanine-amino transferase (ALT), and liver steatosis and fibrosis indices comparable to those seen in bariatric surgery [7,8,9]. ESG offers a reversible technique which has a faster procedure time, a shorter or no hospital length of stay, and a better safety profile when compared to bariatric surgery [10].

Weight loss after ESG is primarily mediated not only through the restriction of stomach volume and changes in the stomach morphology but also significantly through delayed gastric emptying. This latter mechanism plays a crucial role in enhancing satiety, reducing overall caloric intake, and contributing to weight management post-procedure. Therefore, changes in gastric volume and shape and the slowing of gastric emptying after the procedure are thought to be the main determinants of weight loss achieved. Additionally, ESG has been shown to lead to lower levels of ghrelin in addition to earlier satiety and delayed gastric emptying leading to significantly reduced caloric intake and weight loss. However, the effect of the change in gastric dimensions after ESG on the amount of weight loss has not been well-studied to date. The aim of this study was to assess the fluoroscopic measurement of gastric dimensions after ESG as a predictor of weight loss. Specifically, we utilized fluoroscopy to measure post-procedural changes in gastric shape and size (identified as gastric fundus/antrum and fundus/body ratios) to predict the Total Body Weight Loss (TBWL) over time.

## 2. Materials and Methods

### 2.1. Study Population and Follow-Up

Patients who underwent ESG from August 2013 to August 2019 were prospectively enrolled in a patient registry. Between January 2015 and March 2018, all consecutive patients were referred for upper GI fluoroscopy within 7 days after the procedure. Patients were included in this study if they attended their appointment for fluoroscopy and completed at least 12 months of follow-up. All procedures were performed in a single center by the same gastroenterologist (R.S.). This study was approved by the institutional review board at our medical center (IRB Protocol 1510016654).

The inclusion criteria for undergoing ESG procedure included body mass index (BMI) of more than 30 kg/m^2^, and failure of previous noninvasive weight loss measures including pharmacotherapy to achieve a sustainable TBWL of at least 5%. Patients with a BMI of more than 40 kg/m^2^ were included if they refused to undergo bariatric surgery or were deemed not to be surgical candidates. The exclusion criteria included prior bariatric procedures, family history of gastric cancer, history of neoplastic gastric lesions, major mental health disorders as evaluated by a psychologist, coagulopathy, and any significant comorbidities that may interact with deep sedation. Before the decision was made to perform ESG, patients were evaluated by a multidisciplinary team including a gastroenterologist, an endocrinologist, a dietitian, and a psychologist. Patients were not required to stop pharmacotherapy after ESG procedure.

Anthropomorphic measurements and laboratory studies were performed at baseline before the procedure, and during scheduled follow-up visits at 1, 3, 6, and 12 months after the procedure. Afterward, patients were encouraged to continue to have, at minimum, yearly follow-up visits. A phone call or video follow-up was carried out if patients did not attend their follow-up visits. All participants were scheduled to have a follow-up visit with their dietitian after the procedure and were required to continue with lifestyle and diet interventions according to the recommendations of their provider.

### 2.2. ESG Procedure

Data were recorded on the technical details of the ESG, which was performed as described previously [11]. In summary, the procedures were performed in an outpatient endoscopy suite, under either propofol sedation or general anesthesia at the discretion of the anesthesiology provider. The procedures were performed in the left lateral position, using a standard gastroscope (GIF-H190, or GIF-HQ190; Olympus, Center Valley, PA, USA) with CO_2_ insufflation. After mapping anterior and posterior suture lines from incisura to cardia with argon plasma coagulation (APC), a double-channel gastroscope (GIF-2TH180; Olympus) outfitted with the OverStitch suturing device (Apollo Endosurgery, Austin, TX, USA) was used to perform the procedure. During the initial phase of the study, an overtube was used during the suturing; however, with increased experience, the use of overtube was stopped. Full-thickness sutures were placed in two layers from incisura to cardia (sutures are not placed in the fundus), in an either Z or U pattern, to approximate the anterior and posterior walls of the stomach and achieve a tubular reconfiguration and decreased gastric volume. All patients received pre-procedural prophylactic antibiotic (levofloxacin 500 mg intravenously), the sleeve was lavaged with 80 mg of topical Gentamicin at the end of the procedure, and three days of oral antibiotics were given after the procedure. Antiemetics were used before and after the procedure as described in previous studies [10]. Initially, patients were admitted overnight for observation, but, after observing the safety of ESG during the first 11 procedures, the procedure was changed to a same-day discharge after short observation in the post-anesthesia care unit. All patients were restricted to a full-liquid diet for the first two weeks after the procedure as previously described in the literature [12].

### 2.3. Definitions and Outcomes

Weight loss was assessed using Percent Total Body Weight Loss (%TBWL = [(Initial Weight) − (Postop Weight)]/[(Initial Weight)] × 100), and Percent Excess Body weight loss (%EWL = [(Initial Weight) − (Postop Weight)]/[(Initial Weight − ideal body weight)] × 100; ideal body ideal weight is defined by the weight corresponding to a BMI of 25 kg/m^2^) [13].

**Figure 1:**
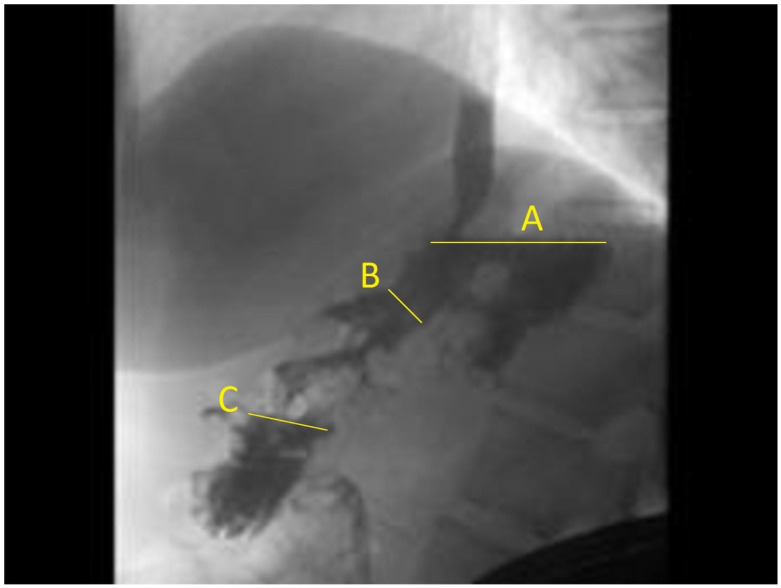
Upper GI fluoroscopy after ESG with measurement of (A) fundus, (B) gastric body, and (C) antrum.

Two independent radiologists who were blinded to patients’ weight loss reviewed all upper GI (UGI) fluoroscopic images. They measured the gastric lumen transverse diameter in three separate areas of fundus, body, and antrum. We measured fundus-to-body and fundus-to-antrum ratios (Figure 1). These ratios were selected, as ESG does not involve suturing the fundus, and, therefore, the fundus diameter could be used to standardize the change in gastric lumen diameter across patients. The decision to adopt this measurement was based on the review of several of our previous cases, which demonstrated its consistency as the most reliable metric. We employ the fundus-to-antrum ratio rather than absolute volumes to provide a more individualized assessment of the gastric configuration. The mean of the two independent measurements was used as the final measurement in the analysis. The primary outcome of this study was achieving a TBWL of 10% or more after ESG at any point during follow-up. The secondary outcomes were achieving five and fifteen percent or more of TBWL after ESG were used in sensitivity analysis. A cut-off of 10% TBWL was chosen based on prior studies showing consistent improvement in metabolic comorbidities associated with obesity by achieving 10% TBWL after endoscopic bariatric therapies (EBTs) [14,15].

### 2.4. Statistical Analysis

Descriptive statistics were reported as means (standard deviation, SD), median (interquartile range, IQR), or counts and proportions. Variables were analyzed using paired Student *t*-test, Chi-squared, and Fisher’s exact tests in univariable analysis. Logistic and linear regressions were used for multivariate analysis. Multilevel mixed-effects logistic regression with fixed effect for time since the procedure and random intercept for individual patients were used to test the linear trend of change in the outcomes after ESG. Multilevel mixed-effects logistic regression was used to build a model in half of patients selected randomly (i.e., building sample) for predicting the probability of achieving a TBWL of 10% or more over time using fluoroscopic measurements as the main variable. These patients were randomly selected irrespective of the date of the procedure to ensure that the analysis would not be biased by the operators’ learning curve over time, allowing for a more accurate assessment of the model’s performance and applicability. Other variables that were evaluated were baseline BMI and their compliance with follow-up visits. This allowed for weight loss variation between study participants. The model was subsequently validated using the remaining half of the cohort (i.e., validation sample), with receiver operating characteristic (ROC) curve discriminant analysis and calibration plot (with 8 deciles of prediction score). The performance of fluoroscopic parameters was also separately evaluated with receiver operating characteristic (ROC) curve analysis and likelihood ratios. All tests are two-tailed with a significance level of alpha = 0.05. All analyses were performed with Stata 13.0 for Windows, StataCorp LP (College Station, TX, USA).

## 3. Results

One hundred and sixty-two consecutive patients were included in the analysis. Table 1 summarizes the study population’s characteristics. In total, 65% of the patients were female, and patients had a mean BMI of 39 ± 6 at baseline. Patients were followed for a mean of 23 months (23.4 ± 18.9, Median 24 months, IQR 6–36 months). Overall, patients had a mean maximum TBWL of 16.5 ± 8.3% during follow-up. Respectively, 92, 75, and 50% of patients achieved a TBWL of 5%, 10%, or 15% or more during follow-up (Table 1). 

On fluoroscopic examination, none of the patients had contrast extravasation after ESG. The mean post-procedural UGI gastric fundus/antrum transverse measurement ratio was 1.2 ± 0.6, and the fundus/body ratio was 3.3 ± 1.1 (Figure 1). 

### 3.1. Prediction Model 

A random sample of 81 patients was included in the building sample to generate models for predicting the %TBWL during follow-up based on the fundus-to-antrum and fundus-to-body ratios measured within 7 days after the procedure. The fundus-to-body ratio was not associated with weight loss during follow-up after adjusting for patients’ compliance and baseline BMI (Table 2). A higher fundus-to-antrum ratio was significantly associated with the primary outcome of a TBWL of 10% or more during follow-up in a multivariable model (OR 2.49, 95% CI 1.31–4.71; *p*-value 0.005). In the sensitivity analysis, a higher fundus-to-antrum ratio was also significantly associated with a TBWL of 15% or more and showed a trend towards an association with a weight loss of 5% or more during follow-up (Table 1). Therefore, the prediction model was built using the fundus-to-antrum ratio in the random building subset of the patients (Table 3).

### 3.2. Validation

**Figure 2:**
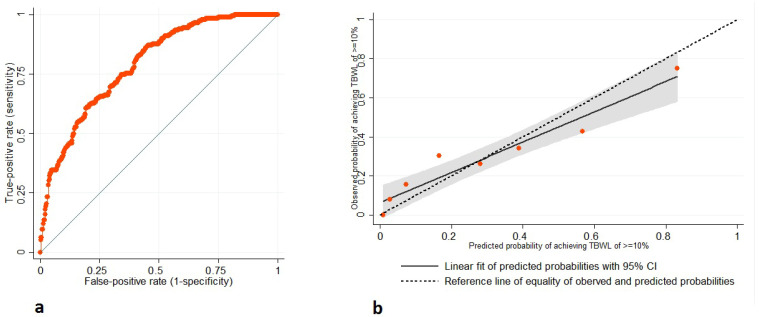
Validation of prediction model based on fundus-to-antrum ratio measured within 7 days after ESG for prediction of total body weight loss (TBWL) of 10% or more during follow-up: (**a**) discrimination, receiver operating characteristic curve (AUC = 0.79, 95%CI 0.75–0.83); and (**b**) calibration plot of observed versus predicted probabilities of achieving TBWL of 10% or more with good agreement between observed and predicted probabilities.

In the discriminant analysis, the prediction score from the multivariable model based on the fundus-to-antrum ratio had an area under the ROC curve of 0.79 (95% CI 0.75–0.83) for predicting a TBWL of 10% or more during follow-up (Figure 2a). The calibration plot showed the appropriate calibration for the agreement between the observed and predicted probabilities of achieving 10% TBWL or more across 8 deciles of predicted probability in the validation sample (Figure 2b). The fundus-to-body ratio of equal to or more than 1.23 as a univariable predictor had a sensitivity of 60.6% and specificity of 61.0% for achieving a TBWL of 10% or more during follow-up, and classified 60.9% of outcomes correctly, with a positive likelihood ratio of 1.6 and a negative likelihood ratio of 0.7.

## 4. Discussion

ESG is effective for achieving significant weight loss with a mean TBWL of nearly 17% observed in this study. We were able to show that the gastric fundus/antrum ratio measured within one week of ESG appears to be a consistent and independent measure with a good accuracy and calibration to predict sustained TBWL during long-term follow-up after the procedure.

Minimally invasive endoscopic techniques, such as ESGs [6,16], have become increasingly common over the past decade leading to alternative management options for patients seeking sustained weight loss. Although lifestyle modifications, medications, and intensive exercise programs are effective in obtaining an initial weight loss of >5%, sustained weight loss in such patients in a general setting has not been significant [2].

ESG offers a minimally invasive approach to obtaining sustained TBWL. The goal of the ESG procedure is to reduce the length and width of the stomach to facilitate TBWL. The procedure requires the use of a stapler or a T-fastener device to create a smaller stomach with a tubular reconfiguration of the gastric lumen. Previously, techniques for ESG utilized a partial thickness suture resulting in dehiscence and weight regain. Modern techniques utilizing full-thickness transmural suturing have been developed and demonstrate a maintained reduction in weight and BMI [6]. Additional fasteners or staples can be applied until the desired lumen size is reached [17].

Prior studies have demonstrated that young age, a high TBWL at one month after ESG, compliance with scheduled visits, and endoscopist experience (>35 ESG cases [11]) were predictors of high TBWL after the procedure [6]. To date, no studies have demonstrated objective post-procedural measurements of the gastric lumen as predictive measures for sustained TBWL. 

We report the post-procedural prediction of sustained TBWL based on the effect of ESG on gastric volume and geometry in terms of the fundus-to-antrum ratio. The gastric fundus-to-antrum ratio measured within one week of ESG appears to be a consistent and independent predictive measure of sustained TBWL in our study. One proposed mechanism for sustained weight loss after ESG appears to revolve around gastric volume with a decreased volume correlating with sustained weight loss [18]. The morphological changes (assessed using MRI in a similar study) have been observed to ultimately result in delayed gastric emptying, driving the weight loss observed following the procedure [19]. Specifically, an enlarged gastric fundus, coupled with a comparatively small body, results in extended food retention within the fundus. This anatomical modification significantly extends the duration of satiety. It is our practice (and the standard practice at many centers) to spare most, if not all, of the fundus above the cardia when performing ESG. Therefore, it is used as a self-reference for each patient to evaluate the change in the gastric anatomy after ESG. However, the changes in gastric hormones and motility have been proposed as important mediators of weight loss after ESG as well. Ghrelin is a multifaceted appetite-regulating hormone secreted mainly by enteroendocrine cells in the stomach fundus and is involved in the stimulatory effects on food intake, fat deposition, and growth hormone release [20]. Prior reviews have suggested that ghrelin requires post-gastric feedback, which may not be regulated through insulin [21]. ESG appears to prevent a change in ghrelin level in postprandial and fasting states as well as prevent a more long-term increase after weight loss which promotes beneficial changes [18]. ESG has also been shown to increase the gastric emptying time, prompting early satiety [14]. Early satiety promotes weight loss due to a decrease in overall caloric intake. It is possible that an increased fundus-to-antrum ratio post-ESG favors an increased emptying time and the differential filling of fundus and delayed passage through antrum. This can lead to significant TBWL through the mentioned hormonal and motility changes in addition to the overall decrease in gastric volume. Importantly, these outcomes are achieved without increasing procedure-related side effects, underscoring the procedure’s safety profile in addition to its efficacy. 

Beyond an attempt at clarifying the physiological basis of weight loss after ESG, our findings offer quality improvement goals in terms of techniques utilized by endoscopists. Focusing on the body of the stomach for suturing to create a larger fundus-to-antrum ratio can potentially lead to better weight loss outcomes and fewer complications. The fundus of the stomach has a thinner wall and is located closer to the diaphragm. It is considered more likely to incur complications such as full-thickness tears and delayed perforations and leaks. By focusing on the body of the stomach during ESG, endoscopists can help decrease complications while improving TBWL.

Moreover, there were a few limitations of the study. Our ESGs were performed by a single bariatric endoscopist, and further studies will be needed to assess the significance of the fundus-to-antrum ratio using different techniques. Further investigation is necessary as well regarding simultaneous hormonal and gastric motility measurements with measurements of gastric size and shape. Furthermore, the utilization of an upper GI fluoroscopy for our measurements limits the ability to quantify gastric volumes after the procedure.

## 5. Conclusions

ESG offers a minimally invasive approach to achieve sustained weight loss. Our findings suggest a specific value for the early post-procedural fundus-to-antrum ratio for prediction of weight loss after ESG. This is imperative in the development of ESG techniques to achieve better results and minimize complications. These results help clarify the underlying mechanisms of weight loss after ESG. Lastly, it can be used during the follow-up of patients, as a non-invasive way to assess changes in the gastric anatomy after ESG, especially in cases of weight loss plateau or weight regain.

## Figures and Tables

**Table 1:** Baseline characteristics of the study population.

Patient Characteristic	Total, *n* = 162	Random Model Building Sample, *n* = 81	Random Validation Sample, *n* = 81
Age, mean (SD), years	46 (13)	48 (12)	44 (13)
Female, *n* (%)	106 (65)	55 (68)	51 (63)
BMI, kg/m^2^, mean (SD)	39 (6)	39 (6)	38 (6)
Fundus-to-antrum ratio, mean (SD)	1.2 (0.6)	1.3 (0.7)	1.1 (0.6)
Fundus-to-body ratio, mean (SD)	3.3 (1.1)	3.5 (1.1)	3.2 (1.0)
TBWL%, mean (SD)	16.5 (8.3)	15.9 (7.8)	17.1 (8.8)
TBWL ≥ 5%, (%)	92	90	94
TBWL ≥ 10%, *n* (%)	75	70	79
TBWL ≥ 15%, (%)	50	48	52

**Table 2:** Association between fundus-to-body and fundus-to-antrum ratio measured immediately after endoscopic sleeve gastroplasty with total body weight loss during follow-up.

Outcome	Fundus-to-Antrum Ratio		Fundus-to-Body Ratio	
	OR (95%CI)	*p*-Value	OR (95%CI)	*p*-Value
TBWL 5% or more	1.93 (0.94–3.97)	0.075	1.02 (0.67–1.55)	0.929
TBWL 10% or more	2.49 (1.31–4.71)	0.005	0.84 (0.58–1.22)	0.361
TBWL 15% or more	2.78 (1.28–6.07)	0.010	0.67 (0.42–1.09)	0.106

**Table 3:** Parameters of the prediction model for predicting total body weight loss of 10% or more during follow-up based on fundus-to-antrum ratio measured immediately after ESG.

Variable	Coefficient	Standard Error (SE)	95% CI
Fundus-to-antrum ratio	0.911	0.326	−0.082 to −0.055
Compliance	2.038	0.410	0.273 to 1.549
Baseline BMI	0.036	0.032	1.234 to 2.843
Time since procedure	−0.069	0.007	−0.027 to 0.099
Intercept	−2.531	1.354	−5.186 to 0.124

## Data Availability

The original contributions presented in the study are included in the article; further inquiries can be directed to the corresponding author.
